# Low-Protein-Fed Chickens Benefit from Probiotic *L. salivarius* and *L. johnsonii* on Performance and Microbiota

**DOI:** 10.3390/ani15223346

**Published:** 2025-11-20

**Authors:** Xiaomei Dong, Xufeng Dou, Hao Tang, Yuanyuan Huang, Guiling Wu, Wei Dong, Hui’e Wang, Haihong Jiao, Yuxia Mei, Min Ren

**Affiliations:** 1 College of Life Science and Technology, Tarim University, Alar 843300, China; 120130023@taru.edu.cn (X.D.);; 2 College of Hydraulic and Architectural Engineering, Tarim University, Alar 843300, China; 3 Key Laboratory of Conservation and Utilization of Biological Resources in the Tarim Basin, Alar 843300, China; 4 Xinjiang Nuoqi Baicheng You Chickens Development Co., Ltd., Aksu 843000, China; 5 College of Animal Science and Technology, Tarim University, Alar 843300, China; 6 College of Life Science and Technology, Huazhong Agricultural University, Wuhan 430070, China

**Keywords:** *Lactobacillu*, low-protein diet, growth performance, gut microbiot

## Abstract

This study aimed to evaluate the combined effects of two probiotic strains, *Lactobacillus salivarius* TRM58163 and *Lactobacillus johnsonii* TRM59525—originally isolated from human infant feces (aged 0–6 months)—on growth performance, antioxidant capacity, liver function, intestinal morphology, and cecal microbiota in 100-day-old Baicheng You chickens fed a low-protein diet. Ten strains isolated from infant feces were initially screened, and eight were assessed in vitro for safety, gastrointestinal tolerance, and antimicrobial activity. TRM58163 and TRM59525 showed the best probiotic potential. A total of 240 Baicheng You chickens (100 days old) were randomly assigned to two groups: The control group was fed a low-protein diet (CLD), while the experimental group had *Lactobacillus salivarius* TRM58163 and *Lactobacillus johnsoni* TRM59525 added to the low-protein diet (LLD, ≥1 × 10^9^ CFU/g feed). Each treatment included four replicates of 30 chickens over 42 days. Probiotic supplementation significantly improved average daily feed intake (ADFI), average daily gain (ADG), and feed conversion ratio (FCR) (*p* < 0.05). It also enhanced antioxidant status, with increased plasma superoxide dismutase activity and reduced malondialdehyde levels. Lower total bilirubin levels indicated improved liver function. The LLD group showed increased ileal villus height and villus-to-crypt ratio. 16S rRNA gene sequencing revealed greater microbial diversity, increased beneficial genera (e.g., *Akkermansia*, *Bacteroides*), and decreased harmful taxa (e.g., *Sutterella*). These microbial shifts were associated with improved growth and metabolic profiles. In conclusion, *L. salivarius* TRM58163 and *L. johnsonii* TRM59525 supplementation improved performance, gut health, and microbial composition, supporting their use as functional probiotics in low-protein poultry diets.

## Simple Summary

The present study isolated bacterial strains with potential probiotic properties from human infant feces (aged 0–6 months) and subsequently evaluated the combined effects of strains *Lactobacillus salivarius* TRM58163 and *Lactobacillus johnsonii* TRM59525 on 100-day-old Baicheng You chickens maintained on a low-protein diet. Supplementation with these two strains increased feed intake and average daily gain (ADG), and improved the feed conversion ratio; furthermore, enhanced antioxidant status and the villus-to-crypt ratio were elevated. The treated birds exhibited greater intestinal microbial diversity, expansion of beneficial taxa, and a reduction in potentially harmful microorganisms. These findings provide preliminary evidence that this probiotic combination enhances production performance and modulates the intestinal microbiota of Baicheng You chickens, demonstrating its potential value as a functional additive for low-protein poultry diets.

## 1. Introduction

Probiotics are defined as live microorganisms which, when administered in adequate amounts, confer health benefits to the host. These beneficial microbes play a pivotal role in modulating gut microbiota and enhancing host immune responses [1]. In modern poultry production, probiotics are increasingly adopted as alternatives to antibiotic growth promoters to support health and performance [2]. Numerous studies have demonstrated that dietary supplementation with probiotics can significantly improve growth performance, strengthen immune function, enhance nutrient utilization, and support intestinal health by favorably modulating the gut microbial community [3,4]. The diversity and stability of the gut microbiota are considered essential for maintaining overall health in poultry [5], as accumulating evidence suggests that a balanced gut microbial ecosystem plays a central role in regulating nutrient metabolism, immune modulation, and disease resistance [6].

Adoption of low-protein (LP) diets represents a promising strategy to address the dual challenges of protein resource scarcity and environmental sustainability in poultry production. LP diets are characterized by reduced crude protein content, supplemented with synthetic amino acids to meet the specific nutritional requirements of poultry, thereby maintaining growth performance and product quality [7]. In addition to alleviating pressure on global protein supplies, LP feeding offers considerable environmental and economic benefits. However, excessive reduction in dietary protein can have detrimental effects on animal health and performance [8,9,10]. Several studies have reported that LP diets may impair intestinal integrity, induce systemic inflammation, and disrupt intestinal morphology, particularly by reducing villus height and compromising nutrient absorption, ultimately resulting in reduced growth performance [11]. These drawbacks can also lead to economic losses for producers. Although strategies such as amino acid and enzyme supplementation have been explored to lessen the negative effects of low-protein diets, research on the use of probiotics for this purpose remains scarce. This represents a significant knowledge gap, particularly given that the mechanisms underlying probiotic functionality under protein-restricted conditions remain poorly understood [12,13,14]. Therefore, evaluating the potential of probiotic supplementation in LP diets is not only timely but essential for supporting poultry health, optimizing nutrient utilization, and promoting sustainable development in the livestock industry.

Among the diverse probiotic genera, *Lactobacillus*, *Streptococcus*, *Lactococcus*, and *Bifidobacterium* have emerged as particularly effective due to their demonstrated health-promoting properties. Notably, *Lactobacillus* species are well recognized for their ability to produce digestive enzymes such as proteases, amylases, lipases, and phytases, thereby enhancing nutrient digestion and absorption [15,16]. Among these, *Lactobacillus salivarius*, a common intestinal inhabitant in humans, pigs, and poultry, has shown considerable promise in improving gut morphology, egg production, microbial balance, and immune regulation, while exhibiting antimicrobial activity [17,18,19]. Similarly, *Lactobacillus johnsonii*, another Gram-positive bacterium prevalent in the gastrointestinal tract, has demonstrated comparable benefits for host health and performance [20,21]. Recent evidence suggests that multi-strain probiotic combinations can provide synergistic effects by colonizing different intestinal regions and complementing functional roles, thereby producing enhanced overall efficacy [22]. Therefore, can the dietary supplementation of *Lactobacillus salivarius* and *Lactobacillus johnsonii* effectively promote poultry growth, enhance nutrient utilization, and improve health status? Whether the effects are more pronounced under special feeding conditions such as low-protein diets warrants further investigation.

Baicheng You Chickens, a native breed with over 300 years of history, originate from Baicheng County in Aksu Prefecture, Xinjiang, China. Known for their distinctive subcutaneous fat distribution [23]. These chickens are prized for their high muscle mass, rich skin fat content, superior flavor, medicinal properties, strong disease resistance, and notable cold tolerance [24]. Despite their desirable traits, Baicheng You Chickens exhibit slow growth and require a prolonged rearing period of 6-8 months before slaughter. The lack of established feeding standards and the high cost of commercial feeds further hinder their productivity and feed efficiency. These challenges not only limit the breed’s commercial viability but also pose a threat to the preservation and sustainable utilization of this valuable genetic resource [25,26].

Despite considerable research on Baicheng You Chickens, there is limited investigation into the application of probiotics in this breed. Specifically, the effects of probiotics on growth performance and cecal microbiota in Baicheng You Chickens under low-protein (LP) feeding conditions remain poorly understood. Moreover, no previous work has explored the combined application of *Lactobacillus salivarius* and *Lactobacillus johnsonii* in this context. Therefore, the present study aimed to evaluate the combined effects of *L. salivarius* TRM58163 and *L. johnsonii* TRM59525—two strains originally isolated from human infant feces—on growth performance, feed conversion efficiency, antioxidant capacity, plasma biochemical parameters, liver function, intestinal morphology, and cecal microbial ecology in Baicheng You Chickens fed a low-protein diet. The overarching goal was to establish a scientific basis for using these Lactobacillus strains as functional feed additives capable of enhancing growth and health in poultry under protein-restricted conditions, thereby contributing to sustainable breeding and efficient resource utilization in both heritage and commercial poultry systems.

## 2. Materials and Methods

### 2.1. Bacterial Isolation and Identification

Collect stool samples from infants aged 0–6 months of the same sex for bacterial isolation. Fresh fecal specimens (2 g) were suspended in 18 mL of sterile distilled water and homogenized at 37 °C with agitation at 200 rpm for 20 min. The homogenate was serially diluted in sterile saline, and 100 µL aliquots of the 10^−4^ and 10^−5^ dilutions were spread in triplicate onto de Man, Rogosa and Sharpe (MRS) agar plates [27]. Following incubation at 37 °C for 24 h under aerobic conditions, colonies exhibiting distinct macroscopic morphologies were selected and streak-purified for a minimum of three consecutive passages to ensure clonal purity. Genomic DNA was extracted from single colonies using a commercial kit according to the manufacturer’s instructions. The 16S rDNA gene was amplified with the universal bacterial primers 27F and 1492R, and gene were purified and sequenced bidirectionally (Sangon Biotech (Shanghai) Co., Ltd., Shanghai, China). Taxonomic assignment was performed by matching through the EzBioCloud 16S database (https://www.ezbiocloud.net/ accessed on 28 August 2024).

### 2.2. In Vitro Evaluation of Probiotic Properties

2 μL of each *Lactobacillus* culture were spotted onto Columbia blood agar and incubated at 37 °C for 48 h; hemolytic activity was judged by the presence or absence of a clear zone around the colony [28]. Antibiotic susceptibility was screened against ten antimicrobial agents by the Kirby-Bauer disk-diffusion method, with inhibition zones measured after 24 h at 37 °C [29]. Acid tolerance was assessed in MRS broth adjusted to pH 2.0 and 3.0, while bile tolerance was tested in media containing 0.2% and 0.3% bile salts; viable counts (CFU) were determined after 24 h at 37 °C [30]. Survival under gastrointestinal conditions was evaluated by exposing the strains to simulated gastric fluid and subsequently to simulated intestinal fluid, enumerating CFU after each step [31]. The antimicrobial activity of cell-free supernatants from each strain against pathogenic bacteria was evaluated using the Oxford cup method; inhibition zones were measured to assess the inhibitory effects on the pathogens [32]. Each LAB strain was inoculated into MRS broth at 2% (*v*/*v*) and incubated statically at 37 °C for 24 h. Optical density at 600 nm (OD_600_) was recorded every 2 h with a spectrophotometer, and growth curves were generated by plotting OD_600_ against time. All experiments were performed in triplicate.

### 2.3. Experiment Design

Baicheng You Chickens (100-day-old) were housed in a closed, environmentally controlled poultry house with automatically regulated temperature, humidity, and ventilation, and provided with 24 h continuous lighting. After being housed for seven days, the chickens were weighed individually, and 240 healthy chickens with uniform weight were selected and randomized into two treatment groups. The control group was fed a LP diet (CLD), while the experimental group had *L. salivarius* TRM58163 and *L. johnsoni* TRM59525 added to the low-protein diet (LLD). The viable bacterial count of each strain was ≥ 1 ×10^9^ CFU/g. Each group had 4 replicates per treatment, and 30 chickens each replicate. The experimental cycle was 42 days. Table 1 shows the composition and nutrient levels of LP.

### 2.4. Animal Experiment Design and Management

This experiment was conducted at the Tarim Animal Disease Diagnosis and Control Engineering Laboratory, Xinjiang Production and Construction Corps. Prior to the study, chicken coops and all feeding equipment were thoroughly cleaned and disinfected. Chickens were housed in three-tier cages, with two chickens per cage. Throughout the trial, chickens had ad libitum access to feed and water. Fecal trays were cleaned twice daily to maintain hygiene, and routine disease prevention procedures were followed. Replicates were treated as experimental units. Body weights were recorded on Days 7 (starter phase: Days 0–7), 22 (grower phase: Days 8–21), and 43 (finisher phase: Days 22–42). Chickens were fasted for 12 h before each weighing to calculate average daily gain (ADG). Daily feed intake was recorded to determine average daily feed intake (ADFI) and feed conversion ratio (FCR) for each phase.

### 2.5. Bacterial Culture Preparation

*L. salivarius* TRM58163 and *L. johnsonii* TRM59525 are protease-producing strains previously isolated by our laboratory through screening for acid and bile salt tolerance, antimicrobial activity, antibiotic susceptibility, and safety. Strain preparation followed a modified version of the method described by Liu et al. [33]. Both strains were inoculated into MRS broth and incubated at 37 °C for 24 h. Bacterial cells were harvested by centrifugation (2000 r/min, 5 min, 4 °C), then mixed with skim milk (Beijing Coolab Tech Co., Ltd., Beijing, China) and lyophilized to form probiotic powder. The powder was stored at 4 °C and administered daily in combination with LP, as described by Li et al. [34]. Viability was assessed biweekly to ensure a viable cell count of 1–2 × 10^9^ CFU/g.

### 2.6. Sampling Procedure

At the end of Weeks 3 and 6, venous blood was randomly collected from two chickens per replicate using heparinized disposable tubes. Samples were centrifuged at 3500 r/min for 15 min (high-speed cryo-centrifuge, Thermo Fisher Scientific, Shanghai, China), and the plasma was stored at −20 °C for biochemical and antioxidant analyses. After blood collection, the chickens were euthanized. Digestive organs-including the proventriculus, gizzard, and pancreas—were excised, blotted dry with absorbent paper, and weighed. The liver, duodenum, jejunum, and a 3 cm segment of the proximal ileum were fixed in 4% paraformaldehyde for histomorphological analysis. Pancreatic and cecal contents were immediately snap-frozen in liquid nitrogen and stored at −80 °C for subsequent assays. The relative organ weight was calculated as follows:

Relative weight of digestive organs (g/kg) = organ weight (g)/live weight of chicken (kg)(1)

### 2.7. Plasma Markers and Pancreatic Trypsin Assay

Plasma levels of total protein (TP), albumin (ALB), blood urea nitrogen (BUN), creatinine (Crea), total bilirubin (TB), aspartate aminotransferase (AST), and alanine aminotransferase (ALT) were measured using a fully automated biochemical analyzer (SMT-120VP, Chengdu Smarter Science and Technology Co., Ltd., Sichuan, China). Plasma diamine oxidase (DAO) activity was quantified using a Diamine Oxidase Assay Kit (Metware Biotechnology Inc., Woburn, MA, USA). Plasma superoxide dismutase (SOD) and malondialdehyde (MDA) levels were measured with an assay kit from Nanjing Jiancheng Bioengineering Institute. For pancreatic trypsin analysis, 1 g of pancreas tissue was homogenized in physiological saline with zirconia dioxide beads using a high-throughput tissue homogenizer (Scientz-48L, Ningbo Scientz Biotechnology Co., Ltd., Ningbo, China) at 60 Hz for 300 s at 4 °C. The homogenate was centrifuged at 3000 r/min for 10 min, and the supernatant was collected. Pancreatic trypsin content was measured using a chicken trypsin ELISA kit.

### 2.8. Intestinal Histology and Microbial Diversity Analysis

Intestinal and liver tissues were fixed, dehydrated, cleared, embedded, sectioned, and stained with hematoxylin and eosin (H&E). Histological observations were performed using an upright light microscope (Eclipse Ci-L, Nikon, Japan). Villus height (V.H.) and crypt depth (C.D.) were measured using Case Viewer 2.4 software, and the villus-to-crypt ratio (V/C) was calculated according to Hosseindoust et al. [35]. For microbial diversity analysis, a total of 32 cecal content samples were collected—16 at Week 3 and 16 at Week 6. Microbial community composition was analyzed by 16S rDNA high-throughput sequencing (Shanghai Majorbio Bio-pharm Technology Co., Ltd., Shanghai, China).

### 2.9. Statistical Analysis

All data was processed using Microsoft Excel, and then SPSS 26.0 software was used for independent sample t-test analysis. The results were expressed as the mean ± the standard error of the mean (SEM). Differences with *p* < 0.05 are considered statistically significant, differences with *p* < 0.01 are considered highly significant, and differences with *p* < 0.001 are considered extremely significant. Origin 2021 software was used for drawing. The significance levels are denoted as follows: * for *p* < 0.05, ** for *p* < 0.01, and *** for *p* < 0.001. Cecal amplicon data was analyzed and plotted on the Majorbio cloud platform (https://cloud.majorbio.com, accessed on 22 November 2024).

## 3. Results

### 3.1. Composition of Intestinal Bacteria and Candidate Probiotic 

**Figure 1:**
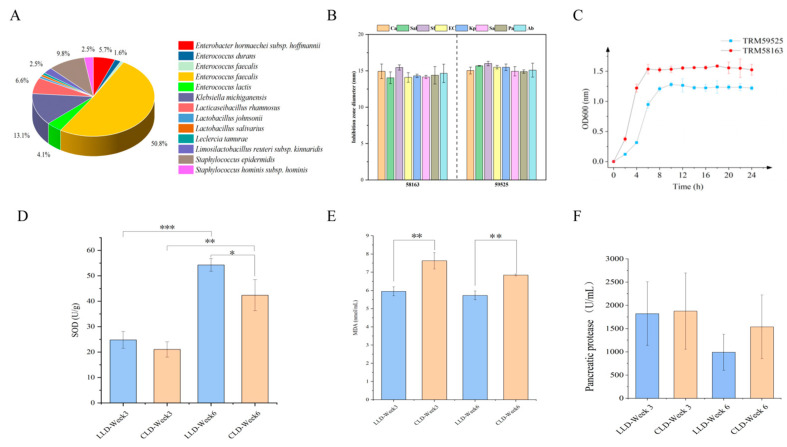
(**A**) Species-level relative abundance of Lactobacillus strains isolated from infant feces. (**B**) Antagonistic activity of *Lactobacillus salivarius* TRM58163 and *Lactobacillus johnsonii* TRM59525 against selected enteric pathogens. (**C**) Growth kinetics of *L. salivarius* TRM58163 and *L. johnsonii* TRM59525 in MRS broth under anaerobic conditions. (**D**) Circulating superoxide dismutase (SOD) concentrations in the CLD and LLD groups at 3 and 6 weeks. (**E**) Plasma malondialdehyde (MDA) levels in the CLD and LLD groups at 3 and 6 weeks. (**F**) Serum trypsin activity in the CLD group relative to the LLD group at weeks 3 and 6. The significance levels are denoted as follows: * for *p* < 0.05, ** for *p* < 0.01, and *** for *p* < 0.001.

A total of 122 bacterial strains were isolated and purified from infant faecal samples. Taxonomic identification, based on 16S rRNA gene sequencing, revealed that 96 isolates belonged to the phylum *Firmicutes* and 25 to *Proteobacteria*, encompassing six genera and thirteen species (Figure 1A). Within this collection, ten isolates were identified as *Lactobacillus* spp., comprising one strain of *L. salivarius* designated TRM58163 (Genbank: PV961220), one *L. johnsonii* TRM59525 (PV961116), and eight strains of *Lacticaseibacillus rhamnosus* designated TRM59538 (PV961127), TRM59542 (PV961130), TRM59546 (PV961133), TRM59549 (PV961136), TRM59554 (PV961141), TRM59560 (PV961146), TRM59568 (PV961153), and TRM59569 (PV961154).

### 3.2. In Vitro Characterization of Probiotic Properties of the Isolates

In vitro assessment of probiotic properties demonstrated that all ten *Lactobacillus* strains were non-hemolytic, confirming their biosafety profile. Antibiotic susceptibility testing revealed that most strains were sensitive to penicillin (PEN), chloramphenicol (C), and ampicillin (AMP). Notably, strains TRM59542 and TRM59554 exhibited resistance to all tested antibiotics, whereas *L. rhamnosus* TRM59568 was susceptible to all agents evaluated (Table S1). Acid and bile salt tolerance assays showed that all strains, except TRM59568 and TRM59569, were capable of growing under acidic conditions (pH 2.0–3.0) and in the presence of bile salts (0.2–0.3%). Among these, *L. johnsonii* TRM59525, *L. salivarius* TRM58163, and *L. rhamnosus* TRM59538 exhibited superior viability under stress conditions (Table S2). Simulated gastrointestinal fluid tolerance was further evaluated in six selected strains. *L. johnsonii* TRM59525, *L. salivarius* TRM58163, and *L. rhamnosus* TRM59549 maintained relatively high viable counts following 3 h of exposure to simulated gastric fluid and subsequent incubation in simulated intestinal fluid (Table S3). Pathogen inhibition assays identified only *L. johnsonii* TRM59525 and *L. salivarius* TRM58163 as effective inhibitors of most tested pathogens (Figure 1B). Synthesizing these findings, *L. johnsonii* TRM59525 and *L. salivarius* TRM58163 demonstrated the most favorable overall probiotic potential and were thus selected as promising candidates. Growth curve analysis confirmed typical sigmoidal growth, with logarithmic phases spanning 0–8 h for TRM59525 and 0–6 h for TRM58163 (Figure 1C).

### 3.3. Growth Performance

Compared to the control low-protein diet (CLD), the low-protein diet supplemented with *L. salivarius* and *L. johnsonii* (LLD) significantly increased the average daily feed intake (ADFI) of Baicheng You Chickens, particularly during the grower and finisher stages, as well as across the entire rearing period (*p* < 0.01). These results suggest that probiotic supplementation with *L. salivarius* and *L. johnsonii* enhances feed intake and appetite. Furthermore, at the starter stage (Days 1–7), the average daily gain (ADG) in the LLD group was significantly higher than that in the CLD group (*p* < 0.05). During the grower and finisher stages and the overall rearing cycle, LLD consistently exhibited significantly higher ADG than CLD (*p* < 0.01), indicating that the probiotic combination effectively promotes growth under low-protein conditions. In terms of feed efficiency, LLD showed a significantly lower feed-to-gain ratio than CLD at the starter stage, and this improvement was even more pronounced throughout the entire experimental period, where the difference in feed conversion ratio (FCR) reached high statistical significance (*p* < 0.001). Collectively, these findings demonstrate that *L. salivarius* and *L. johnsonii* supplementation enhances growth performance and feed efficiency in Baicheng You Chickens fed a low-protein diet (Table 2).

### 3.4. Plasma Markers, Antioxidant Capacity and Trypsin

No significant differences were observed between the LLD and CLD groups in the plasma levels of amylase, triglycerides (TG), total protein (TP), blood urea nitrogen (BUN), and alanine aminotransferase (ALT). At Week 3, total bilirubin (TB) levels were significantly higher than those at Week 6 (*p* < 0.05), whereas albumin (ALB) levels were significantly lower (*p* < 0.05). Additionally, in the CLD group, aspartate aminotransferase (AST) levels were significantly elevated at Week 6 compared to Week 3 (*p* < 0.05). These findings suggest that probiotic supplementation may have a stabilizing effect on hepatic function, particularly regarding bilirubin and albumin metabolism (Table S4).

Superoxide dismutase (SOD) activity increased significantly in both groups over time (*p* < 0.001), with consistently higher values observed in the LLD group throughout the rearing cycle, suggesting enhanced antioxidant capacity (Figure 1D). Malondialdehyde (MDA) levels were significantly lower in the LLD group compared to the CLD group at both Weeks 3 and 6 (*p* < 0.05) (Figure 1E), indicating reduced lipid peroxidation. In contrast, pancreatic trypsin levels were lower in the LLD group than in the CLD group at Weeks 3 and 6, further indicating potential modulation of digestive enzyme activity by probiotic supplementation (Figure 1F).

### 3.5. Relative Weight of Digestive Organs and Histomorphology

The relative weights of digestive organs were higher in the LLD group than in the CLD group, although the differences were not statistically significant (*p* > 0.05). From Week 3 to Week 6, the relative weight of digestive organs increased in the LLD group. In contrast, within the CLD group, the relative weight of the gizzard increased at Week 6, whereas the weights of the pancreas and proventriculus declined, though these changes were also not significant (*p* > 0.05) (Table 3).

**Figure 2:**
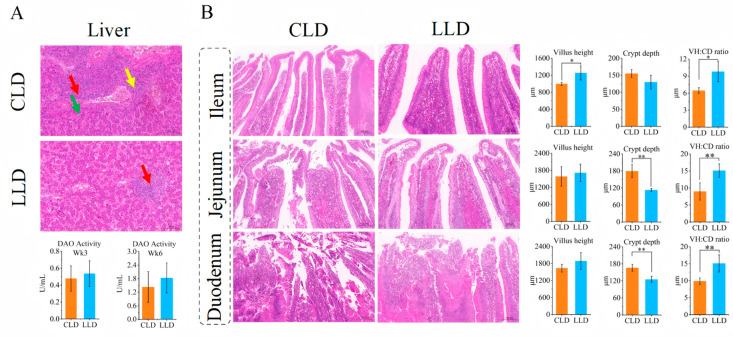
Histological Examination. (**A**) Liver paraffin sections and hematoxylin—eosin (H&E) staining of the CLD and LLD groups at Week 3. Levels of diamine oxidase in the liver were measured at Weeks 3 and 6. In the CLD group, there was nuclear fragmentation and dissolution, with numerous inflammatory cells infiltrating (red arrow). Mild perivenular fibrosis was commonly seen in liver tissue (yellow arrow), and a large amount of eosinophilic material was present in the hepatic sinusoids (green arrow). In the LLD group, focal hepatocyte necrosis disappeared, and a small number of inflammatory cells were observed to infiltrate (red arrow). (**B**) Paraffin sections and H&E staining of the small intestine (duodenum, jejunum, and ileum) of the CLD and LLD groups at Week 3. Villus height, crypt depth, and villus-to-crypt ratio (V/C) were assessed in different intestinal segments. * and ** indicate *p* < 0.05 and *p* < 0.01.

Histological analysis of liver tissue revealed markedly reduced pathological changes in the LLD group compared to CLD. Specifically, LLD exhibited less hepatocyte necrosis and fibrosis, along with overall alleviation of hepatic lesions. Moreover, plasma diamine oxidase (DAO) activity was elevated in the LLD group relative to CLD at both time points, implying improved intestinal barrier function (Figure 2A). In terms of intestinal morphology, the ileal villus height (V.H) in the LLD group was significantly higher than in the CLD group (*p* < 0.05), and increases were also observed in the jejunal and duodenal V.H. Conversely, crypt depth (C.D) in the ileum, jejunum, and duodenum was significantly lower in the LLD group (*p* < 0.01). Consequently, the villus height-to-crypt depth ratio (V/C) was significantly increased at all intestinal sites in the LLD group (*p* < 0.05), indicating enhanced absorptive capacity and improved intestinal integrity (Figure 2B).

### 3.6. Effects on the Cecal Microbiota

After sequencing, assembly, and quality control, a total of 1,120,058 valid sequences were obtained, with an average sequence length of 1452 bp. Taxonomic annotation identified the following classifications: 2 domains, 3 kingdoms, 20 phyla, 38 classes, 57 orders, 105 families, 238 genera, 397 species, and 762 operational taxonomic units (OTUs). OTU clustering was conducted based on a 97% sequence similarity threshold. Venn diagram analysis revealed 638 shared OTUs in Week 3 and 662 in Week 6. Additionally, 4 and 9 unique genera were identified in LLD at Week 3 (L3) and Week 6 (L6), respectively, while 11 and 7 unique genera were found in CLD at Week 3 (C3) and Week 6 (C6).

**Figure 3:**
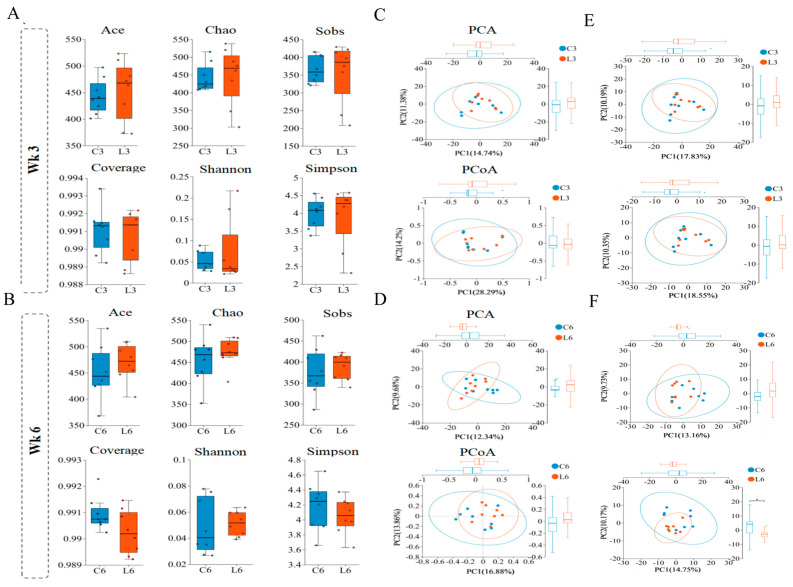
Cecal Microbiota Diversity. Comparison of α-diversity indices (Ace, Chao 1, Shannon, Simpson, sobs, and coverage) of cecal microbiota between CLD and LLD groups. (**A**) At Week 3 (Wk3). (**B**) At Week 6 (Wk6). β-diversity analysis of cecal microbiota between CLD and LLD groups. (**C**) At Week 3 (Wk3), the OTU level. (**D**) At Week 6 (Wk6), the OTU level. (**E**) At Week 3 (Wk3), genus level and species level. (**F**) At Week 6 (Wk6), genus level and species level. * indicate *p* < 0.05.

Alpha diversity analysis showed no significant differences between LLD and CLD in ACE, Chao1, Shannon, Simpson, Sobs, and coverage indices at the OTU level, suggesting that *L. salivarius* and *L. johnsonii* supplementation did not markedly alter overall cecal microbiota structure. However, the median ACE and Shannon indices were slightly higher in the LLD group at both time points, indicating a trend toward increased microbial richness and diversity (Figure 3A,B). Principal component analysis (PCA) based on OTU profiles revealed significant separation between LLD and CLD at Week 6 (*p* < 0.05), while principal coordinate analysis (PCoA) demonstrated distinct clustering between groups at both Week 3 and Week 6 (*p* < 0.05) (Figure 3C,D). Further PCA at the genus and species levels indicated significant differences between LLD and CLD at Week 3 (*p* < 0.05), and highly significant differences at the species level were observed at Week 6 (*p* < 0.01) (Figure 3E,F).

### 3.7. Microbial Composition Analysis 

**Figure 4:**
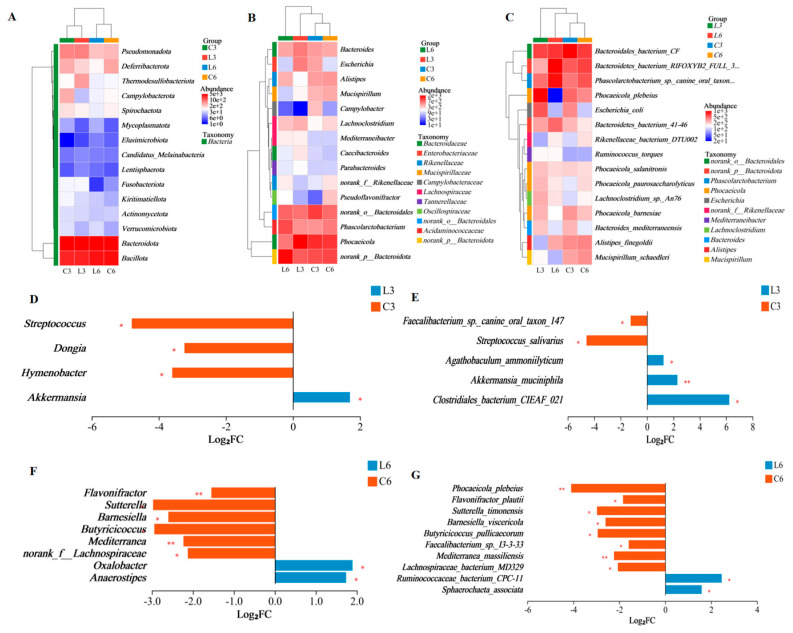
Cecal microbiota profiling. (**A**–**C**) Relative abundance at the phylum, genus, and species levels, respectively. (**D**,**E**) Differential taxa (genus and species) between CLD and LLD groups At Week 3. (**F**,**G**) Differential taxa (genus and species) At Week 6. The significance levels are denoted as follows: * for *p* < 0.05, ** for *p* < 0.01.

*Bacteroidota* was identified as the dominant phylum in the cecal microbiota of Baicheng You Chickens. It accounted for 54.88% and 54.32% of the microbial community in the LLD and CLD groups, respectively, at Week 3, and 55.02% and 50.34%, respectively, at Week 6. The second most abundant phylum was *Bacillota*, which represented 29.23% (LLD) and 28.03% (CLD) at Week 3, and increased to 39.40% (LLD) and 38.58% (CLD) by Week 6 (Figure 4A). Genus-level analysis revealed increased abundances of *Phocaeicola*, *Bacteroides*, and *Lachnoclostridium* in the LLD group compared to CLD at Week 3. At Week 6, *norank_p__Bacteroidota* and *Phascolarctobacterium* were more abundant in LLD than in CLD. Significant differences in microbial composition at the genus level were observed in the LLD group at Week 6 (Figure 4B). At the species level, dominant taxa in the CLD group included *Bacteroidales bacterium* CF, *Bacteroidetes bacterium* RIFOXYB2 FULL 397, and *Phascolarctobacterium* sp. canine oral taxon 149. In contrast, the LLD group was dominated by *Phocaeicola plebeius*, *Bacteroidales bacterium* CF, and *Phascolarctobacterium* sp. canine oral taxon 149 (Figure 4C).

### 3.8. Significant Differential Microbial Analysis 

**Figure 5:**
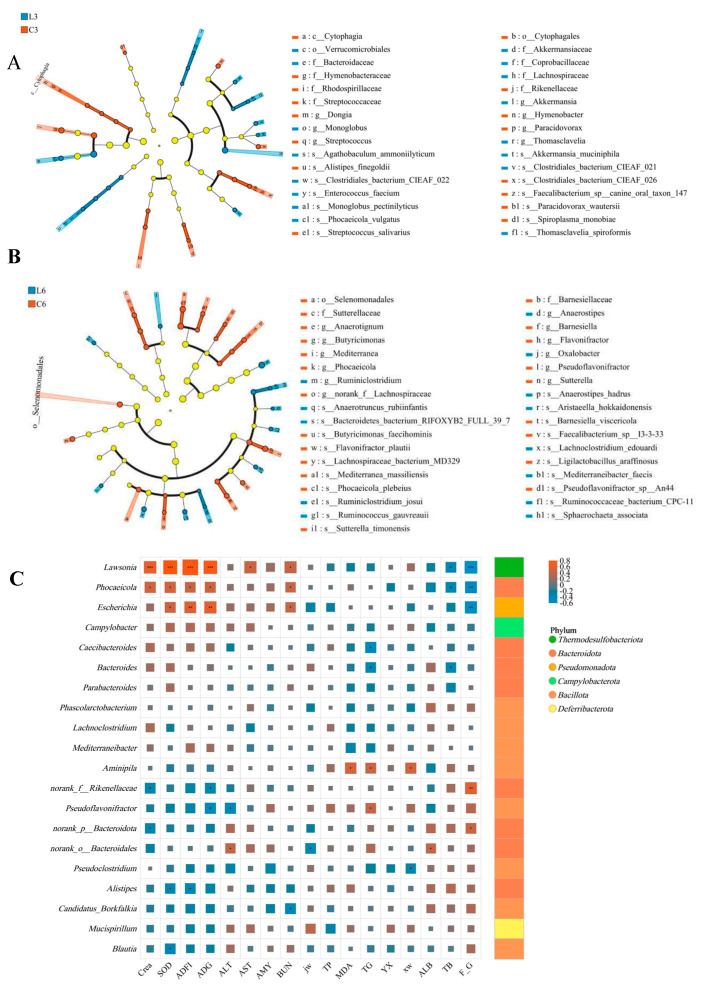
Cecal microbiota profiling. (**A**,**B**) LEfSe cladograms highlighting discriminant features at weeks 3 and 6 (LDA score > 2.0). (**C**) Spearman correlation heatmap between differential taxa, plasma biomarkers, and feed-efficiency indices. The significance levels are denoted as follows: * for *p* < 0.05, ** for *p* < 0.01, and *** for *p* < 0.001.

Inter-group difference analysis showed that on Week 3 of feeding, compared with CLD, LLD had a higher abundance of *Akkermansia* and lower abundances of *Streptococcus*, *Dongia*, and *Hymenobacter* at the genus level (*p* < 0.05) (Figure 4D). At the species level, the abundance of *Clostridiales bacterium* CIEAF_021 (*p* < 0.05) and *Akkermansia muciniphila* (*p* < 0.01) were higher in LLD, while the abundances of *Faecalibacterium* sp. canine oral taxon 147 and *Streptococcus salivarius* were lower (*p* < 0.05) (Figure 4E). At Week 6 of rearing, the abundance of *Oxalobacter* and *Anaerostipes* was higher in LLD as compared to CLD, while the abundance of *Sutterella*, *Barnesiella*, *Butyricicoccus*, and *norank_f__Lachnospiraceae* was lower (*p* < 0.05). The abundance of *Flavonifractor* and *Mediterranea* was lower at the genus level in LLD as compared to CLD (*p* < 0.01) (Figure 4F). At the species level, the abundance of *Sphaerochaeta associata* and *Ruminococcaceae bacterium* CPC-11 was higher, whereas the abundance of *Flavonifractor plautii*, *Sutterella timonensis*, *Barnesiella viscericola, Butyricicoccus pullicaecorum, Faecalibacterium* sp. I3-3-33, and *Lachnospiraceae bacterium* MD329 was lower (*p* < 0.05). Additionally, the abundance of *Phocaeicola plebeius* and *Mediterranea massiliensis* was lower in LLD as compared to CLD (*p* < 0.01) (Figure 4G). Linear discriminant analysis effect size (LEfSe) (LDA > 2, *p* < 0.05) was used to identify the bacterial taxa with significant inter-group differences from the genus to phylum level. Linear discriminant analysis effect size showed that in the 3rd week of feeding, compared with CLD, in LLD, the relative abundance of *Akkermansia* and *Verrucomicrobiales* was upregulated, while in CLD, the relative abundance of *Cytophagales* and *Cytophagia* was higher (Figure 5A). In the 6th week of feeding, the abundance of *Anaerotignum* and *Anaerostipes* was higher in LLD, while the abundance of *Selenomonadales* and *Sutterellaceae* was higher in CLD (Figure 5B).

### 3.9. Correlation Analysis

Spearman correlation analysis was conducted to examine the associations between cecal microbiota composition and key physiological indicators, including growth performance, plasma biochemical markers, antioxidant capacity, and relative digestive organ weights in the experimental chickens. *Lawsonia* showed significant positive correlations with average daily feed intake (ADFI), average daily gain (ADG), superoxide dismutase (SOD), creatinine (Crea), blood urea nitrogen (BUN), and aspartate aminotransferase (AST) (*p* < 0.05), and was negatively correlated with feed conversion ratio (FCR) and total bilirubin (TB) (*p* < 0.05). Similarly, *Phocaeicola* was positively associated with ADFI, ADG, SOD, Crea, and BUN (*p* < 0.05), and negatively correlated with FCR and TB (*p* < 0.05). Escherichia also showed positive correlations with ADFI, ADG, SOD, and BUN, and a negative correlation with FCR (*p* < 0.05). In contrast, *Bacteroides* was significantly positively correlated with TB and triglycerides (TG) (*p* < 0.05). *Pseudoflavonifractor* was positively correlated with TG, but negatively correlated with ADG and alanine aminotransferase (ALT) (*p* < 0.05). Unclassified_f__*Oscillospiraceae* exhibited positive correlations with FCR and malondialdehyde (MDA), and negative correlations with ADFI, ADG, SOD, and Crea (*p* < 0.05). Additionally, *Alistipes* was positively associated with both ADFI and SOD (*p* < 0.05), while *norank_p__Bacteroidota* was positively correlated with FCR and negatively correlated with Crea (*p* < 0.05) (Figure 5C).

## 4. Discussion

*Lactobacillus* supplementation has been demonstrated to improve both the colonization of beneficial bacteria in the intestines of animals and the host–gut ecological balance, thereby increasing feed intake and promoting animal growth [36]. Adding *L. salivarius* mixture in chicken feed increases the weight and feed conversion ratio in broiler chickens, improving the gut histomorphology [18]. This experiment found that adding *L. salivarius* and *L. johnsonii* to Baicheng You Chickens’ low-protein feed significantly enhanced their average daily feed intake, weight gain, and feed conversion rate. Digestive organ index was also ameliorated. Yao et al. studied the growth patterns of Baicheng You Chickens with different plumage colors [37]. They found that roosters and hens reach growth peaks at 10 and 12 weeks of age, respectively. The Von Bertalanffy model best described their growth, with a maximum weekly weight gain of 108.46 g (15.49 g daily). In this study, 100-day-old Baicheng You Chickens were fed low-protein feed with *L. salivarius* and *L. johnsonii* added after their growth peak. Their average daily gain (16.09 g) still exceeded that of chickens on a normal diet (15.49 g), showing that *L. salivarius* and *L. johnsonii* can boost Baicheng You Chickens’ growth. Previous experiments showed that *L. salivarius* and *L. johnsonii* can produce proteases. Chen’s study suggested that proteases from *Bacillus subtilis* break down feed proteins into small molecules for yeast fermentation, enhancing broiler growth [38,39]. The improved growth performance may result from the synergistic interaction between the two Lactobacillus strains and the proteases they produce. Zavelinski et al. reported that adding protease to the diet can improve the growth performance of broiler chickens [40]. This study indicates that the proteases from *L. salivarius* and *L. johnsonii* likely enhance the decomposition of macromolecular nutrients in feed, improving nutrient absorption efficiency. This in turn boosts the utilization efficiency of low-protein feed and has a more positive effect on the growth performance of Baicheng You Chickens.

The use of low-protein (LP) diets has become an essential nutritional strategy in animal husbandry to reduce feeding costs and environmental nitrogen excretion [13]. However, LP diets may also lead to disturbances in protein metabolism, hepatic dysfunction, and increased renal burden. Plasma biochemical markers serve as sensitive indicators of nutrient metabolism and overall physiological health [9,41]. Previous studies have demonstrated that *Lactobacillus* supplementation can mitigate the adverse effects of LP diets by modulating protein metabolism and supporting liver function. For instance, *Lactobacillus* has been shown to reduce plasma AST and ALT levels, thereby improving hepatic health [42]. In the present study, dietary supplementation with a combination of *L. salivarius* and *L. johnsonii* significantly increased plasma total protein (TP) and albumin (ALB) levels, while reducing blood urea nitrogen (BUN) and creatinine (Crea) levels in Baicheng You Chickens. These findings suggest that *L. salivarius* and *L. johnsonii* effectively enhances protein digestion and utilization while alleviating renal metabolic load. Moreover, the activities of AST and ALT, as well as total bilirubin (TB) concentrations, were significantly reduced in the LLD group. Histopathological analysis further revealed improved liver structure, characterized by reduced inflammatory infiltration and hepatocellular necrosis, indicating strong hepatoprotective effects of *L. salivarius* and *L. johnsonii* supplementation. These results collectively highlight the potential of *L. salivarius* and *L. johnsonii* to compensate for the nutritional limitations of LP diets through enhancement of liver function and protein metabolism.

Oxidative stress is another critical factor affecting animal health, and antioxidant capacity serves as a key indicator of physiological resilience [43]. The antioxidant properties of *Lactobacillus* are well documented. Research indicates that *Lactobacillus* supplementation elevates plasma activities of antioxidant enzymes such as superoxide dismutase (SOD) and glutathione peroxidase (GSH-Px) while concurrently lowering malondialdehyde (MDA), a key indicator of lipid peroxidation [44,45]. Consistent with these findings, our results showed that *L. salivarius* and *L. johnsonii* supplementation significantly increased plasma SOD activity and decreased MDA levels in Baicheng You Chickens, suggesting enhanced systemic antioxidant capacity and reduced oxidative damage.

Intestinal health is also a pivotal factor influencing poultry growth performance and immune status. Emerging evidence has shown that *Lactobacillus* enhances intestinal morphology, thereby improving nutrient absorption and gut integrity [36,46]. Notably, lactic acid bacteria have been shown to increase villus height (V.H.), reduce crypt depth (C.D.), and improve the villus height-to-crypt depth ratio (V/C), an established indicator of gut absorptive capacity and mucosal health [47]. In the present study, *L. salivarius* and *L. johnsonii* supplementation significantly increased ileal villus height, reduced crypt depth, and improved the V/C ratio, demonstrating enhanced intestinal structural integrity. Although no significant changes were observed in the villus height of the jejunum and duodenum, the positive trend showing greater villus height in the experimental group compared to the control group suggests that *Lactobacillus* may exert specific effects in different regions of the intestine. This could be attributed to differences in physiological function, local immune responses, or microbial community composition across intestinal segments. Additionally, the observed reduction in crypt depth may reflect an increased enterocyte renewal rate, which has been linked to improved gut barrier function and inhibition of pathogen colonization [48]. Overall, our findings confirm that *L. salivarius* and *L. johnsonii* supplementation improves liver and intestinal health, enhances antioxidant capacity, and mitigates the limitations of LP feeding. Future studies should investigate the immunomodulatory and metabolic regulatory mechanisms underlying these effects to further elucidate the role of *Lactobacillus* in poultry gut health and systemic physiology.

A balanced cecal microbiota is essential for maintaining animal health, serving as a critical interface between host metabolism and environmental nutrient exchange. It plays a direct role in regulating nutrient digestion, absorption, and overall gut health [49]. As the intestinal segment harboring the highest microbial density in poultry, the cecum holds substantial research significance. Previous studies have shown that *Firmicutes*, *Bacteroidetes*, and *Proteobacteria* are the predominant phyla in the cecal microbiota of broiler chickens [50]. Probiotic supplementation, including *L. salivarius*, has been reported to beneficially modulate gut microbial communities and promote intestinal health [16,51]. In the present study, dietary inclusion of *L. salivarius* and *L. johnsonii* significantly altered the cecal microbial composition in Baicheng You Chickens, enhancing both microbial diversity and community structure. In particular, increased species evenness and inter-group differentiation were observed, indicating a more stable and functionally diverse microbial ecosystem. Our findings also confirmed that *Bacteroidota* and *Bacillota* were the dominant phyla in the cecum, which aligns with results reported in previous studies [33,52,53].

These phyla are known to play critical roles in carbohydrate metabolism, short-chain fatty acid (SCFA) production, and modulation of host immunity [54,55]. suggesting that *L. salivarius* and *L. johnsonii* supplementation may improve host metabolic and gut health through microbiota-mediated mechanisms. This study showed a significant increase in *A. muciniphila* abundance in the experimental group, which is associated with improved metabolic and inflammatory profiles. *A. muciniphila* contributes to intestinal homeostasis by enhancing barrier function, modulating immune metabolism, and secreting functional proteins such as Amuc_1100 and P9. The experimental group also showed higher levels of beneficial genera (*Bacteroides*, *Phascolarctobacterium*) and lower levels of harmful taxa (*Sutterella*), which supports nutrient absorption, enhances SCFA production, and reduces inflammation. In contrast, *Sutterella* produces IgA proteases that impair mucosal immunity and are linked to ulcerative colitis [56,57]. In addition, species like *Clostridium butyricum* may inhibit intestinal tumor development via regulation of the Wnt signaling pathway [58]. Overall, *L. salivarius* and *L. johnsonii* supplementation improved the cecal microbiota by increasing beneficial and reducing harmful bacteria, consistent with findings by Monika et al. and Cai et al. [53,59]. Further studies should explore their mechanisms and long-term effects on host metabolism and immunity.

Correlation analysis in this study revealed that *L. salivarius* and *L. johnsonii* supplementation significantly altered the cecal microbiota composition in Baicheng You Chickens, correlating with host health markers. Specifically, Bacteroides showed a negative correlation with plasma TB and TG levels, likely due to SCFA metabolic pathways [60]. *Bacteroides* plays a key role in carbohydrate metabolism by degrading complex polysaccharides into monosaccharides, which are further metabolized into SCFAs. These metabolites regulate lipid metabolism and immune function, including inhibiting cholesterol synthesis via HMG-CoA reductase [61,62]. Additionally, *Phocaeicola* was negatively correlated with ADFI and ADG, likely due to its role in intestinal mucosal protection and immunomodulation [63]. *Phocaeicola* enhances gut barrier function and immune regulation through SCFAs and polysaccharide metabolites [64]. *Lawsonia*, typically associated with gut inflammation [65], was positively correlated with ADFI and ADG in this study. This discrepancy may be due to strain specificity, host health, and rearing conditions. In conclusion, *L. salivarius* and *L. johnsonii* supplementation significantly impacted host health and growth performance by modulating the cecal microbiota composition, highlighting its importance in gut health research and its potential to improve economic outcomes.

## 5. Conclusions

Results indicated that among the ten *Lactobacillus* strains isolated from infant feces, *L. salivarius* TRM58163 and *L. johnsonii* TRM59525 demonstrated superior in vitro probiotic properties, including enhanced acid and bile salt tolerance, resistance to simulated gastrointestinal fluids, and potent antimicrobial activity, when compared to the other strains. Based on these findings, Baicheng You chickens were supplemented with these two strains at a dose of 1 × 10^9^ CFU/g of feed for a duration of 42 days. The results indicate that incorporating *L. salivarius* and *L. johnsoni* improves ADG and ADFI and improves the FCR. Additionally, these strains enhance antioxidant capacity, liver health, and gut tissue condition, while increasing the diversity of cecal microbiota and the abundance of beneficial bacteria. Importantly, this research shows that adding specific *Lactobacillus* can boost poultry growth and health without raising feed protein levels. These findings offer innovative strategies for using LP in poultry feed, reducing rearing costs, lowering nitrogen emissions, and enhancing animal welfare. Future studies should further investigate the effects of these *Lactobacillus* strains on different poultry breeds and their practical application in commercial settings.

## Figures and Tables

**Table 1:** Composition and nutrient levels of basal diets for Baicheng You Chickens (air-dry basis, %).

Item	
Ingredients, % ^1^	
Corn	70
Soybean meal	15
Wheat bran	10
Premix1	5
Total	100
Nutritional level ^2^	
ME (MJ/Kg)	12.60
CP (%)	13.21
EE (%)	3.31
CF (%)	2.40
Ca (%)	1.09
TP (%)	0.55

^1^ The premix provides the following per kg of diets: VA 160–290 KIU, VD3 65–100 KIU, VE 480 mg, VK3 40 mg, VB1 37 mg, VB2 113 mg, VB6 70 mg, VB12 0.64 mg, D—biotin 5 mg, D—pantothenic 202 mg, folic acid 25 mg, nicotinic acid 721 mg, Fe 960–2880 mg, Cu 168–500 mg, Mn 1760–3000 mg, Zn 1440–2400 mg, Se 5–10 mg, choline oxide 5400 mg, Met 22,160 mg. ^2^ CP, EE, CF, Ca, TP were calculated values, while the others were measured values.

**Table 2:** The influence of diet supplementation with *Lactobacillus salivarius* and *Lactobacillus johnsonii* on the growth performance of Baicheng You Chickens.

Items	Treatment	Starter	Grower	Finisher	Overall
		0–7 d	8–21 d	22–42 d	0–42 d
ADFI (g)	LLD	48.32 ± 1.37	70.19 ± 2.87	81.57 ± 1.60	66.69 ± 1.76
	CLD	37.24 ± 3.64	46.92 ± 1.95	56.67 ± 0.31	46.94 ± 1.82
	*p*-value	0.046	0.003	0.003	0.001
ADG (g)	LLD	11.67 ± 0.86	16.74 ± 1.26	19.86 ± 0.90	16.09 ± 0.82
	CLD	7.62 ± 1.74	8.79 ± 0.76	10.57 ± 0.17	8.99 ± 0.85
	*p*-value	0.022	0.002	<0.001	<0.001
FCR	LLD	4.15 ± 0.14	4.20 ± 0.16	4.11 ± 0.08	4.15 ± 0.03
	CLD	4.93 ± 0.34	5.35 ± 0.12	5.36 ± 0.07	5.22 ± 0.15
	*p*-value	0.020	0.001	<0.001	0.005

**Table 3:** The influence of diet supplementation with *Lactobacillus salivarius* and *Lactobacillus johnsoni* on the relative weights of digestive organs in Baicheng You Chickens.

Items	LLD	CLD	*p*-Value		
			LLD (Week 3 vs. Week 6)	CLD (Week 3 vs. Week 6)	LLD vs. CLD
Pancreas					
Week 3	2.88 ± 0.78	2.73 ± 0.68	0.44	0.846	0.670
Week 6	3.52 ± 2.07	2.65 ± 0.92			0.303
Proventriculus					
Week 3	4.99 ± 1.19	4.93 ± 0.86	0.270	0.308	0.913
Week 6	5.57 ± 1.64	4.43 ± 0.90			0.072
Gizzard					
Week 3	37.49 ± 8.03	34.92 ± 6.44	0.232	0.741	0.492
Week 6	40.05 ± 9.67	36.12 ± 8.24			0.396

## Data Availability

Raw reads of bacterial 16S rDNA gene sequencing are available in the NCBI Sequence Read Archive database (Accession Number: PRJNA1215258). Information can be made available from the authors upon request.

## Supplementary Materials

The following supporting information can be downloaded at: https://www.mdpi.com/article/10.3390/ani15223346/s1, Table S1: Sensitivity of Lactic Acid Bacteria to antibiotics and hemolytic assay. Table S2: Acid and bile salt tolerance of *Lactobacillus*. Table S3: Viable counts of *Lactobacillus* strains in simulated gastrointestinal fluids. Table S4: Impact of diet supplementation with *Lactobacillus salivarius* and *Lactobacillus johnsoni* on plasma biochemical indicators of Baicheng You Chickens.

## Abbreviations

The following abbreviations are used in this manuscript:

CLD	The Control group was fed a Low-protein Diet
LLD	The group had *Lactobacillus salivarius* TRM58163 and *Lactobacillus johnsoni* TRM59525 added to the Low-protein Diet
LP	Low-Protein
ADG	Footnote explaining Average Daily Feed Intake
ADFI	Footnote explaining Average Daily Gain
FCR	Footnote explaining Feed to Gain Ratio
L3	The LLD group at Weeks 3
L6	The LLD group at Weeks 6
C3	The CLD group at Weeks 3
C6	The CLD group at Weeks 6
