# Supplementary material for: Low-Protein-Fed Chickens Benefit from Probiotic L. salivarius and L. johnsonii on Performance and Microbiota
Source: Animals (Basel). 2025 Nov 20;15(22):3346. doi: 10.3390/ani15223346 (PMC12649553; doi:10.3390/ani15223346)
Supplement: Supplementary file 1 [file animals-15-03346-s001.zip › animals-3948928-supplementary.pdf]

Table S1 Sensitivity of Lactic Acid Bacteria to antibiotics and hemolytic assay.

Susceptibility	Antibiotics										Hemolytic
	MY	CTR	AMP	CI	C	TET	GEN	SXT	E	PEN	
TRM58163	I	S	I	I	S	I	R	R	I	S	-
TRM59525	S	I	I	R	S	T	R	R	S	S	-
TRM59538	I	I	S	R	S	S	I	R	S	S	-
TRM59546	I	I	S	I	S	S	I	R	S	S	-
TRM59549	R	S	S	I	R	I	R	R	R	S	-
TRM59560	I	I	S	I	S	S	I	R	S	S	-
TRM59568	I	I	S	I	S	S	I	I	I	S	-
TRM59569	I	I	S	I	I	S	R	R	S	S	-

Note:Ciprofloxacin(5ug/disk),Ceftriaxone（40 ug/disk）,Ampicillin（10 ug/disk),Erythromycin (15 ug/disk),Chloramphenicol (30 ug/disk),Penicillin(10 ug/disk),Tetracycline (30 ug/disk),Gentamicin (10 ug/disk),Lincomycin (2 ug/disk),Cotrimoxazole (25 ug/disk).

R: Drug resistance; I: Neutral sensitivity; S: Sensitivity; -: No hemolytic ring

Table S2 Acid and bile salt tolerance of *Lactobacillus*

Strain No.	Survival percentage at different pH values				Survival percentage at different times and bile salt concentrations			
	Initial pH2.0	Final pH2.0	Initial pH3.0	Final pH3.0	Initial 0.2%	Final 0.2%	Initial 0.3%	Final 0.3%
TRM59538	1.66×10^{8}	7.20×10^{7}	1.40×10^{8}	4.93×10^{7}	4.33×10^{6}	1.83×10^{5}	1.00×10^{6}	1.67×10^{5}
TRM59549	8.21×10^{8}	5.08×10^{7}	9.65×10^{8}	4.10×10^{7}	1.59×10^{8}	3.57×10^{7}	2.03×10^{7}	1.13×10^{6}
TRM59560	3.00×10^{7}	2.10×10^{6}	8.74×10^{8}	1.39×10^{7}	5.25×10^{7}	3.20×10^{6}	1.50×10^{7}	9.00×10^{4}
TRM59525	1.09×10^{9}	9.70×10^{8}	1.06×10^{9}	7.39×10^{7}	1.15×10^{9}	2.53×10^{8}	1.38×10^{8}	7.33×10^{7}
TRM59546	2.05×10^{8}	7.84×10^{7}	7.79×10^{8}	7.70×10^{6}	1.90×10^{8}	4.33×10^{7}	7.50×10^{7}	1.30×10^{6}
TRM58163	9.27×10^{8}	8.31×10^{8}	9.02×10^{8}	7.80×10^{7}	8.05×10^{8}	6.83×10^{7}	1.19×10^{8}	2.00×10^{7}
TRM59568	-	-	-	-	-	-	-	-
TRM59569	-	-	-	-	-	-	-	-

Table S3 Viable counts of *Lactobacillus* strains in simulated gastrointestinal fluids.

Strain No	Gastric Juice 0h	Gastric Juice 3h	Intestinal Fluid 4h
TRM59538	2.97×10^{8}	1.40×10^{7}	3.15×10^{6}
TRM59549	1.45×10^{8}	2.90×10^{7}	9.25×10^{6}
TRM59560	1.00×10^{7}	1.00×10^{6}	2.50×10^{5}
TRM59525	6.73×10^{8}	7.60×10^{7}	2.63×10^{7}
TRM59546	3.96×10^{8}	1.30×10^{7}	1.80×10^{6}
TRM58163	6.59×10^{8}	3.35×10^{7}	2.22×10^{7}
TRM59568	-	-	-
TRM59569	-	-	-

Table S4 Impact of diet supplementation with *Lactobacillus salivarius* and *Lactobacillus johnsoni* on plasma biochemical indicators of Baicheng You Chickens

Item	LLD [10]	CLD [11]	*P*-value		
			LLD (Week 3 vs. Week 6)	CLD (Week 3 vs. Week 6)	LLD vs CLD
AMY [1] (U/L)					
Week 3	264.67±58.11	326.14±84.40	0.547	0.607	0.574
Week 6	221.57±36.58	278.17±29.48			0.264
TB [2] (μmol/L)					
Week 3	8.95±1.12	6.35±0.67	0.003	0.662	0.12
Week 6	3.03±1.00	7.13±1.22			0.028
TG [3] (mmol/L)					
Week 3	0.43±0.05	0.46±0.06	0.331	0.440	0.687
Week 6	0.51±0.06	0.53±0.06			0.779
ALB [4] (g/L)					
Week 3	14.28±1.28	17.71±1.46	0.048	0.125	0.836
Week 6	16.81±0.31	11.78±0.91			0.001
TP [5] (g/L)					
Week 3	42.97±2.48	41.28±4.79	0.162	0.206	0.768
Week 6	48.78±2.94	48.30±1.91			0.895
BUN [6] (mmol/L)					
Week 3	1.57±0.33	1.62±0.29	0.846	0.362	0.901
Week 6	1.50±0.15	1.32±0.15			0.403
Crea [7] (μmol/L)					

Week 3	12.03±1.06	17.00±5.17	0.070	0.490	0.320
Week 6	14.98±0.83	12.65±0.75			0.121
AST[8] (U/L)					
Week 3	272.25±15.99	245.57±11.87	0.603	0.019	0.214
Week 6	251.57±34.73	354.71±34.63			0.057
ALT[9] (U/L)					
Week 3	8.00±0.95	10.80±1.66	0.880	0.173	0.180
Week 6	8.14±0.40	8.00±0.63			0.848

[1] Footnote explaining Amylase.

[2] Footnote explaining Total Bilirubin.

[3] Footnote explaining Triglyceride.

[4] Footnote explaining Albumin.

[5] Footnote explaining Total Protein.

[6] Footnote explaining Urea Nitrogen.

[7] Footnote explaining Creatinine.

[8] Footnote explaining Aspartate Aminotransferase.

[9] Footnote explaining Alanine Aminotransferase.

[10] Footnote explaining the group fed a low-protein diet supplemented with lactic acid bacteria.

[11] Footnote explaining the group fed a low-protein diet.
